# Characterization of *Triatoma infestans* (Klug, 1834) (Hemiptera: Heteroptera, Reduviidae) from Residual Foci in the States of Bahia and Rio Grande do Sul, Brazil, Using Geometric Morphology

**DOI:** 10.3390/insects14040325

**Published:** 2023-03-28

**Authors:** Solange Peixoto, Tiago Belintani, Dayse Rocha, Felipe Fontes, Natália Frota, Cleber Galvão

**Affiliations:** 1 Laboratório Nacional e Internacional de Referência em Taxonomia de Triatomíneos, IOC, Fiocruz, Rio de Janeiro 21041-250, Rio de Janeiro, Brazil; 2 Instituto de Biologia, Universidade Estadual de Campinas, Monteiro Lobato, Campinas 13083-859, São Paulo, Brazil; 3 School of Pharmaceutical Sciences, São Paulo State University (Unesp), Araraquara 14800-903, São Paulo, Brazil; 4 Programa de Pós-Graduação em Saúde e Ambiente, Universidade Tiradentes, Aracaju 49032-490, Sergipe, Brazil

**Keywords:** *Triatoma infestans*, residual foci, geometric morphometric, Chagas disease

## Abstract

**Simple Summary:**

Foci of *Triatoma infestans* were found in the states of Rio Grande do Sul and Bahia, Brazil. The objective of the present work is to evaluate the morphometric differences between populations of residual and wild focus of *T. infestans* through head geometric morphometry. It is possible to show significant morphometric differences between the studied populations. Furthermore, it is possible to establish a morphometric relationship between these populations and to list hypotheses about the distribution and maintenance of residual foci of *T. infestans* in Brazil.

**Abstract:**

*Triatoma infestans* is one of the main vectors of Chagas disease in Latin America. Although the species is under control in most Latin countries, it is still necessary to maintain epidemiological surveillance. The present study aims to characterize *T. infestans* populations from residual foci in Bahia and Rio Grande do Sul, Brazil, comparing them with natural populations in Argentina and Bolivia. For this, we adopt the geometric morphometry of the heads. It is possible to report the morphometric variety of the studied populations. In addition, we show that the size of the heads contributes to the differentiation between populations, while the shape has less power to discriminate groups. Furthermore, we show that some natural populations have morphometric proximity to residual populations, suggesting a relationship between these triatomines. Our data do not support the origin of residual populations but demonstrate the importance of new studies with other techniques to understand the dynamics of distribution and reintroduction of these vectors in Brazilian territory.

## 1. Introduction

The subfamily Triatominae (Hemiptera, Reduviidae) is made up of hematophagous insects, and all the described species are potential vectors of the protozoan *Trypanosoma cruzi* (Chagas, 1909), the etiological agent of Chagas disease (CD) or American trypanosomiasis (WHO) [1].

*Triatoma infestans* (Klug, 1834), started to occupy the domestic niche in the Bolivian Inter-Andean valleys as long ago as the Precambrian period [2]. In the 20th century, *T. infestans* occupied three distinct areas of dispersal, one isolated by the Andes mountain range (Chile), and the other two separated by regions where climatic conditions were adverse for the species. One of these is made up of the countries Argentina, Paraguay, Peru, and Uruguay, and the other is made up of the southern regions of Brazil (Paraná, Rio Grande do Sul, and Santa Catarina), the southeast region (Rio de Janeiro and São Paulo), and the northeast region (Minas Gerais and Pernambuco) [3].

The Andes Mountains and the climatic conditions of the Andean regions form natural barriers that hampered the process of active dispersion of *T. infestans* [4]. These factors corroborate with the hypothesis of passive dispersion caused through human actions. In the south-central region of Brazil, the dispersion of *T. infestans* occurred after housing expansion in the region. It is probable that the area of continuous dispersion from Bolivia to southern Brazil facilitated the entry of *T. infestans* into Rio Grande do Sul and later São Paulo and Minas Gerais [4]. The intense traffic of mules and donkeys during the gold and coffee cycle in the 18th century between these regions facilitated the process of dispersion in the Brazilian territory [4].

The first hypothesis for the dispersion of *T. infestans* was the Andean hypothesis, which suggests the Bolivian plateau as the place of origin of the dispersion [5,6,7]. However, the description of the presence of wild *T. infestans* in the Bolivian and Argentina Gran Chaco [8,9] led Piccinali et al. (2009, 2011) [10,11] to propose the Argentina Gran Chaco as the center of origin and dispersion of *T. infestans.*

Systematic actions for the chemical control of *T. infestans* populations, established in Brazil in 1975, implemented by the CD Control Program (PCDCh), through the Southern Cone Countries Initiative, and launched in 1991 [6,12,13], led Brazil to obtain certification of interruption of CD transmission in 2006 through the near-elimination of its main domiciled vector, *T. infestans,* granted by the Pan American Health Organization/World Health Organization (PAHO/WHO). Despite the significant impact in relation to vector, transfusion, and congenital transmissions [8,9,11], the risk of CD transmission persists because of the existence of autochthonous species, some of them at risk, such as *Triatoma rubrovaria* (Blanchard, 1843) and *Panstrongylus lutzi* (Berg, 1879). Despite the certification obtained by Brazil in 2006, residual foci of *T. infestans*, a synanthropic species and the main vector of Chagas disease for many years, were recently found in Rio Grande do Sul and Bahia, leading to the hypotheses that resistance to the insecticide used is associated with intrinsic characteristics of the vector, lack of adequate training in insecticide formulation, and migration of specimens from nearby locations [14]. Based on these factors, the current study aimed to comparatively analyze, through geometric morphometry, the residual foci of *T. infestans* in Rio Grande do Sul and Bahia with specimens from Argentina and Bolivia, aiming to characterize the populations of *T. infestans* of these states with the populations from Argentina and Bolivia.

## 2. Materials and Method

### 2.1. Specimens and Identification

**Figure 1:**
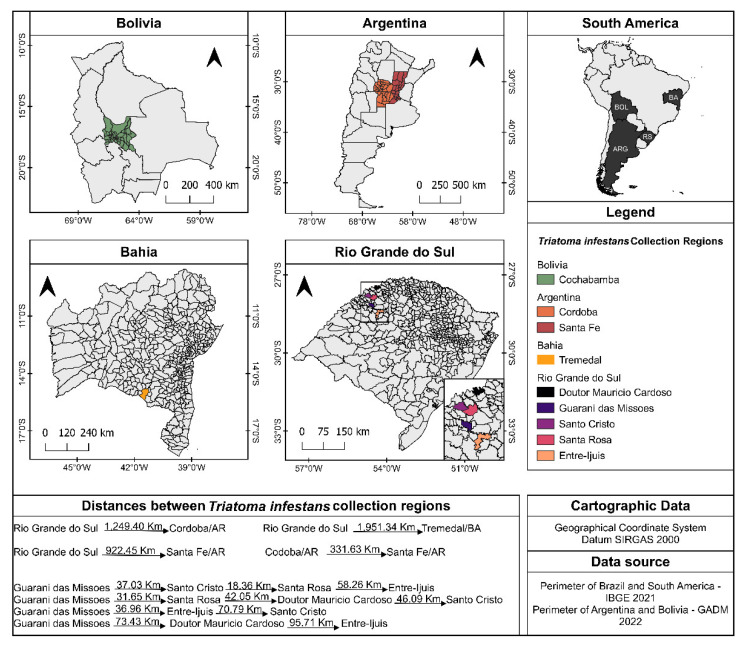
Distribution of the occurrence of *T. infestans* in Argentina, Bolivia, and residual foci in Brazil in the states of Bahia and Rio Grande do Sul.

Triatomines from the following regions were used: Argentina/Cordoba Argentina/Santa Fé; Bolivia/Cochabamba; Bahia (BA)/Brazil; and Rio Grande do Sul (RS)/Brazil (Figure 1). The specimens from the foci used in the study come from the northwest region of the state of Rio Grande do Sul and the municipality of Tremedal, state of BA, Brazil. For the development of the work, specimens and populations of *T. infestans* kept in the Triatomine insectary of the National and International Laboratory of Reference in Taxonomy of Triatomines of the Oswaldo Cruz Institute—Fiocruz, Rio de Janeiro, Brazil were used. The insects from the residual foci of Tremedal, Bahia (BA 2010) came from the Public Health Entomology Laboratory—Faculdade de Saúde Pública—USP (Table 1). These colonies were identified in this study as TI24, TI25, TI26, TI27, TI28, TI131, TI156, TI165, and TI169. The specimens were identified from descriptions and identification keys [15]. Table 1 provides information about the populations studied.

### 2.2. Sampling

A total of 245 heads of male specimens of *T. infestans* were used in the study. The quantity of each population was distributed as follows: TI24 (n = 27); TI 25 (n = 28); TI 26 (n = 30); TI27 (n = 26); TI28 (n = 34); TI131 (n = 30); TI156 (n = 18); TI165 (n = 23); and TI169 (n = 29).

### 2.3. Obtention of Images and Landmarks

Geometric morphometry (GM) was used to evaluate variations in the shape and size of the heads of adult males from *T. infestans* populations. The methodological procedure was the same for all analyses presented in this study. The heads were photographed and digitized using a Leica DMC2900 microscope (Leica Microsystems, Buffalo Grove, IL, USA) with a 3.1 megapixel digital camera (Leica Microsystems) and LAS software (Leica Application Suite United States). The images followed the same settings, aiming to standardize the capture [15].

**Figure 2:**
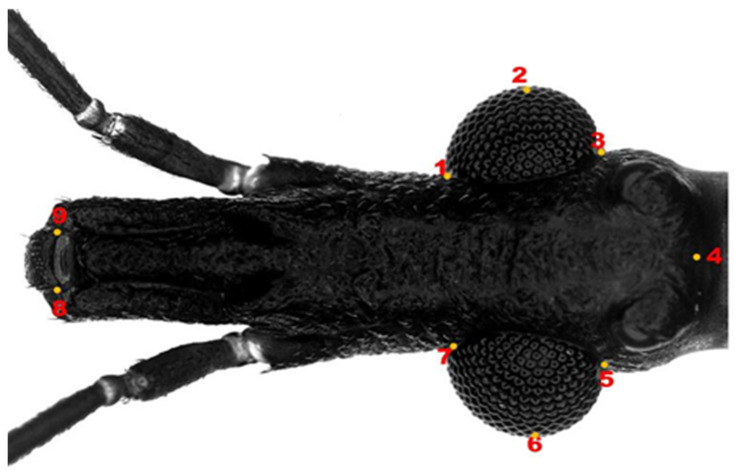
Image of the head of an adult male of *T. infestans*, with marking of the nine type II anatomical landmarks, which were used as coordinates. **1**–**3**: Eye region; **4**: Head size; **5**–**7** Eye region; **8**–**9** Clypeus region.

Eight reference points were selected for the generated matrix (Figure 2). The landmarks were selected as in Oliveira et al. (2017) [16], collected and processed with the TPS package utilities: TPSdig 2.3.2 and TPSutil 1.81 [17,18]. The reference landmarks of all populations were collected three times, seeking to minimize collector effects [17,18]. The order of introduction of the anatomical landmarks were the same for all specimens, as this is a requirement to establish homology of structures. Raw coordinates are used for generalized Procrustes analysis (GPA) in MorphoJ [19]). GPA is a statistical analysis method that is performed to delete all information related to size, position, and orientation [17,18]. Subsequently, the generated matrix is projected onto a Euclidean space to generate a set of scores: partial warps [20]. An average setting, known as a “consensus”, is calculated and allows the average variation between the raw data to be determined [20]. All additional statistics were performed using Procrustes residues to analyze differences in size and shape.

### 2.4. Size and Shape Variables

A factorial map was generated using MorphoJ 1.07a [18] by the general patterns of morphological variation in the multidimensional data obtained with principal component analysis (PCA) using the Procrustes covariance matrix. Coordinates were obtained after the GPA of the original reference coordinates. Procrustes ANOVA [18] was adopted to assess shape variations using MorphoJ 1.07a [18] and to infer differences between groups [20]. To determine the size variables, the isometric estimator defined as centroid size (CS) was used [21]. The CS is derived from raw coordinate data [22] generated in MorphoJ 1.07a [18]. Mahalanobis distances between pairs of populations were calculated for shape measurements and their significance was assessed using a nonparametric permutation-based test (10,000 replicates, Table 2) in MorphoJ 1.07a [18]. Furthermore, a dendrogram was constructed with Mahalanobis distance data, using PAST v.3.25 [23].

### 2.5. Evaluation of Variations between Groups

To determine the relationships between populations (nine regions and three countries), canonical variable analysis (CVA) was performed, using MorphoJ 1.07a [18]. Multivariate statistics were performed using Procrustes coordinates [24]. The CVA was performed associated with a resampling method (10,000 repetitions). A factor map of the first and second canonical factors was generated in MorphoJ 1.07a [18].

## 3. Results

From the collection of nine morphometric landmarks from 245 images and the heads of adult males of the *T. infestans* species, it was possible to demonstrate the morphological relationships of the residual foci in Brazil with the populations of Argentina and Bolivia.

### 3.1. Analysis of Size and Shape Variables between Populations of Nine Localities

**Figure 3:**
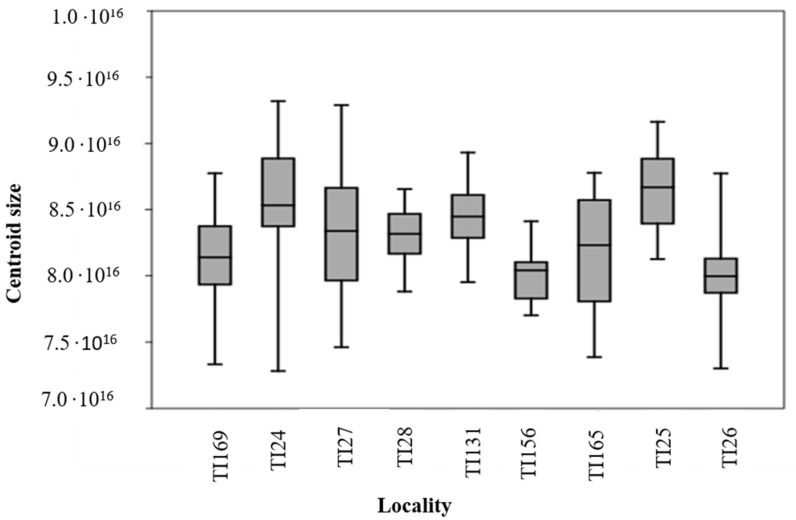
Box diagram of the centroid size of *T. infestans* populations from different localities: TI24 (Guarani das Missões/RS/BR); TI25 (Argentina/Cordoba); TI26 (Argentina/Sta. Fe); TI27 (Entre Ijuís/RS/BR); TI28 (Sto. Cristo/RS/BR); TI131 (Sta. Rosa/RS/BR); TI156 (Sta. Rosa/residual foci/RS/BR); TI165 (Cochabamba/Bolívia); TI169 (Tremedal/Residual foci/BA).

The size variables estimated through the CS show different size means between the studied populations (Figure 3). All recovered averages were significant (*p* < 0.001).

The populations of *T. infestans* from Rio Grande do Sul, Brazil, TI124, TI27, TI28, and TI131, are larger than TI156. Likewise, the two populations of Argentina, TI25 and TI26, show differences in CS, with TI25 being higher. The population of Santa Rosa, Rio Grande do Sul (TI156) and Argentina (TI26) show approximate values. The population from Cordoba, Bolivia has a different metric from the other populations, as well as TI169 from Bahia, Brazil. The size relationship can be described as: TI24 > TI27 > TI25 > TI131 > TI165 > TI26 > TI168 > TI28 > TI156 (Figure 3).

The Mahalanobis distance is a useful distance estimator for determining similarity between samples. Our results allow us to estimate the distances between populations evaluated pairwise (Table 2). The Argentine populations TI25 and TI26 present relatively approximate values, showing proximity between these populations. The population of Rio Grande do Sul, TI24, in relation to the other populations, is close to the Argentine populations (*p* ˂ 0.001). Unlike other Brazilian populations, Bahia and Rio Grande Sul differ significantly from the Argentine specimens and show a relative proximity to TI65 from Argentina. In summary, the populations that present the most discrepant Mahalanobis distance metric are the two Argentinean populations.

**Figure 4:**
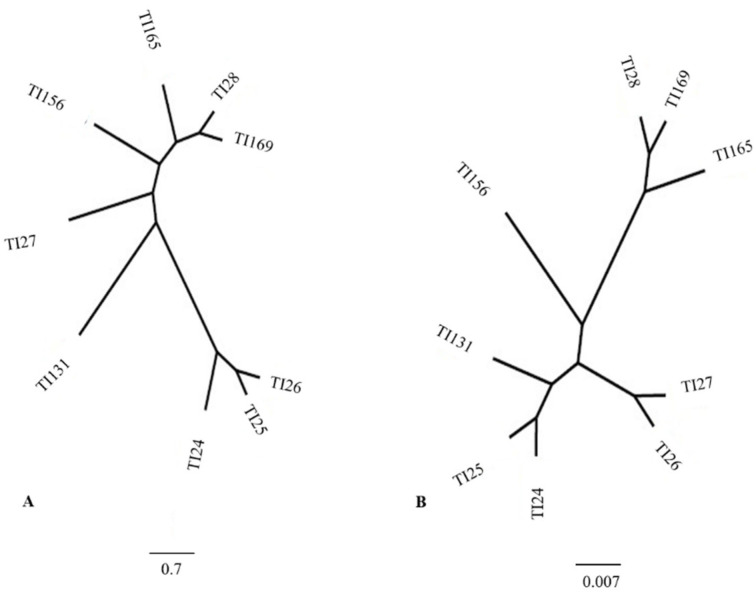
Dendrograms generated after generalized Procrustes analysis among populations from different localities. (**A**) Dendograma of the distance Mahalanobis (**B**) Dendograma of the distance Procrustes. TI24 (Guarani das Missões/RS/BR); TI25 (Argentina/Cordoba); TI26 (Argentina/Sta. Fe); TI27 (Entre Ijuís/RS/BR); TI28 (Sto. Cristo/RS/BR); TI131 (Sta. Rosa/RS/BR); TI156 (Sta. Rosa/Residual foci/RS/BR); TI165 (Cochabamba/Bolívia); TI169 (Tremedal/Residual foci/BA). BR-BRAZIL, RS-Rio Grande do Sul.

The dendrogram retrieved from the Mahalanobis distance data and the Procrustes distance data clearly illustrates the results (Figure 4). The Procrustes distance is a useful method for comparing shape, and, in this study, we built a similarity dendrogram through the retrieved data. Both dendrograms clearly show the close relationship between populations. Populations TI24, TI25, and TI126 form a clade (Figure 4). Populations from Bahia (TI169), Rio Grande do Sul (TI28), and TI165 from Bolivia form a second clade. Populations TI27, TI131, and TI156 from Rio Grande do Sul do not constitute a clade; however, they are closer to the second clade (TI169, TI128, and TI165).

### 3.2. Principal Component Analysis between Populations of Nine Localities

**Figure 5:**
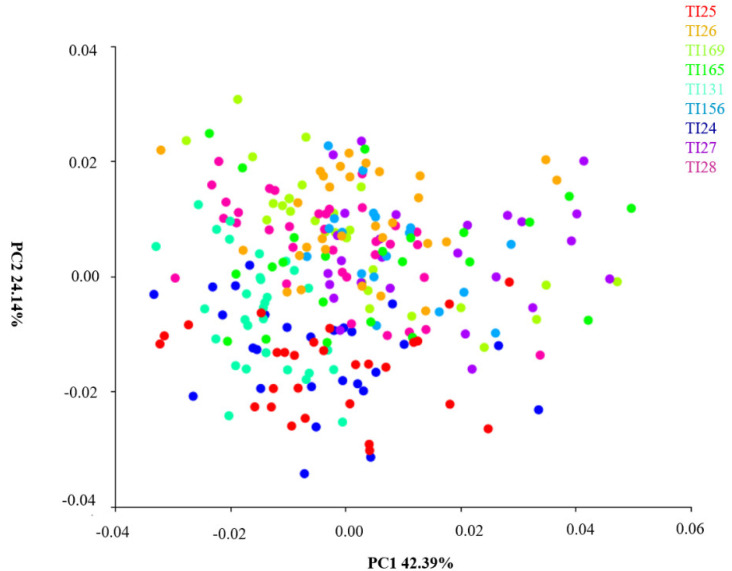
Principal component analysis (PCA) of *T. infestans* populations from different localities. TI24 (Guarani das Missões/RS/BR); TI25 (Argentina/Cordoba); TI26 (Argentina/Sta. Fe); TI27 (Entre Ijuís/RS/BR); TI28 (Sto. Cristo/RS/BR); TI131 (Sta. Rosa/RS/BR); TI156 (Sta. Rosa/residual foci/RS/BR); TI165 (Cochabamba/Bolívia); TI169 (Tremedal/residual foci/BA). BR-BRAZIL, RS-Rio Grande do Sul.

Principal component analysis (PCA), which is defined as an exploratory analysis based on the averages of each population, was able to identify morphological patterns among populations of *T. infestans* (Figure 5).

Principal components explain 100% of the shape variables. The PCA of *T. infestans* populations from different localities showed the following variables: the first principal component (PC1) accounted for 42.39% of the variations, the second component explained 24.14%, and together they accounted for 66.53% of *T. infestans* form variables. The disposition of the ellipses in the space of the Cartesian plane shows an overlapping of the ellipses indicating few differences between the studied forms (heads). Even with similar shapes, head length has greater power of discrimination among the studied specimens.

### 3.3. Canonical Variance Analysis between Populations of 9 Localities

**Figure 6:**
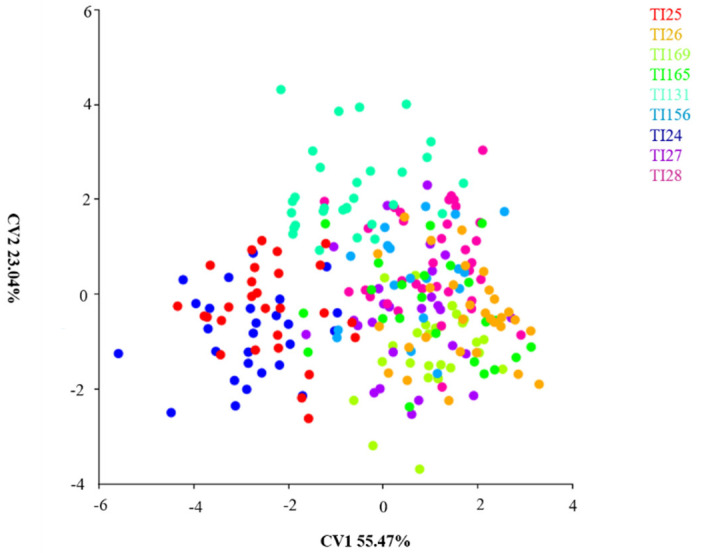
Canonical variance analysis of *T. infestans* populations from different localities. TI24 (Guarani das Missões/RS/BR); TI25 (Argentina/Cordoba); TI26 (Argentina/Sta. Fe); TI27 (Entre Ijuís/RS/BR); TI28 (Sto. Cristo/RS/BR); TI131 (Sta. Rosa/RS/BR); TI156 (Sta. Rosa/residual foci/RS/BR); TI165 (Cochabamba/Bolívia); TI169 (Tremedal/residual foci/BA). BR-BRAZIL, RS—Rio Grande do Sul.

The CVA was used to discriminate groups across datasets. The first component (CV1) was responsible for 55.47% of the shape variations and the second component explained 23.04% of the variation; together they accounted for 78.51% of shape variables in *T*. *infestans* populations from different localities (Figure 6). The space of the Cartesian plane groups the populations TI24 and TI25. The Bolivian population (TI165) is separated from the other study species that appear overlapping, showing little potential for discrimination (Figure 6).

## 4. Analysis of Size and Shape Variables between Populations of Different Regions

To better understand the morphometric diversity of *T. infestans*, we evaluated the populations by grouping them into four groups distributed in Argentina and Bolivia, and two populations from Brazil, Bahia and Rio Grande do Sul, respectively. The objective was to characterize the morphometric patterns of geographically close populations.

### 4.1. Centroid Size between Populations of Different Regions

**Figure 7:**
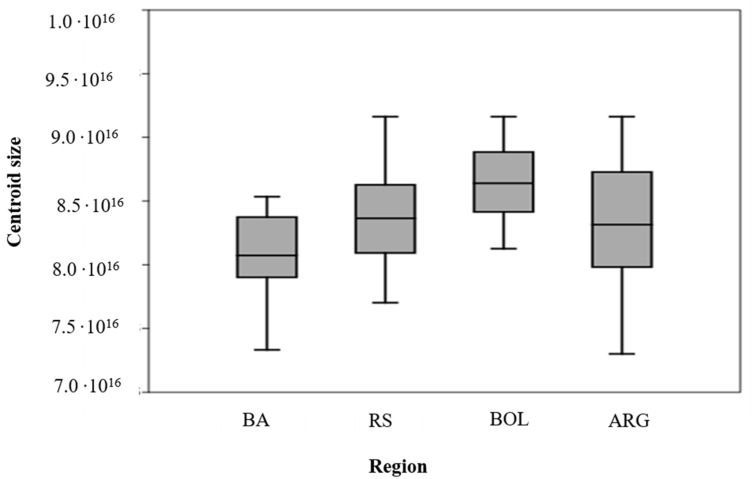
Box diagram of the centroid size of *T. infestans* populations from different regions BA/Bahia/Residual Foci (TI169); RS/Rio Grande do Sul (TI24, TI27, TI28, TI131, TI156 Residual foci); Bolivia/Cochabamba (TI165), Argentina/ARG (Córdoba) (TI25); Argentina/ARG/Santa Fé (TI26).

The CS averages obtained from the GPA show the size variables between populations from different regions. The highest averages of size were *T. infestans* from Bolivia. Populations of *T. infestans* from Argentina and Rio Grande do Sul show approximate values, while *T. infestans* from Bahia shows the lowest average. The size relationship can be described as: *T. infestans* Bolivia > *T. infestans* Argentina (Córdoba/Santa Fe) > *T. infestans* Rio Grande do Sul > *T. infestans* Bahia. The size variation is illustrated by the box plot (Figure 7). In summary, differences in centroid size (CS) between the different populations of Argentina, Bolivia, and Brazil were possible (Figure 7).

Mahalanobis distance values retrieved from show morphometric dissimilarity in size between male heads (Table 3). All values are statistically supported (<0.001). The values show that the populations of the Argentine group are more distant, with less difference with the Rio Grande do Sul group, Brazil. Bolivia’s population is far from all populations. The population of Bahia, Brazil, according to the sampled data, is different from the other Brazilian population (Table 3).

**Figure 8:**
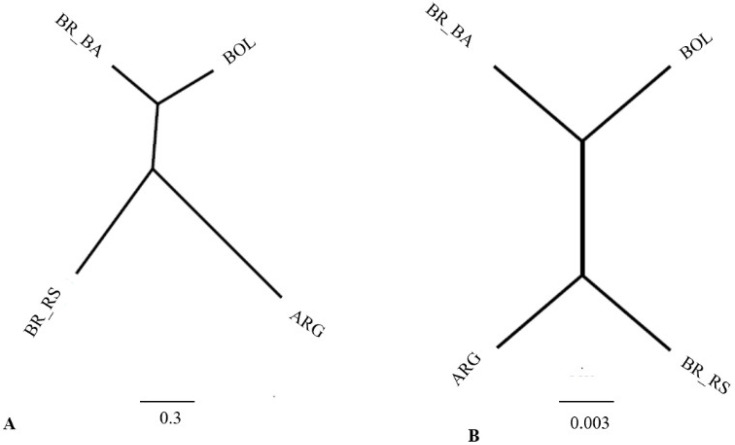
Dendrograms generated after generalized Procrustes analysis among populations from different regions. (**A**) Mahalanobis (**B**) Procrustes. ARG/Argentina (Córdoba) (TI25); ARG/Argentina (Santa Fé) (TI26); BA/Bahia/Brazil/Residual Foci (TI169); Bolivia/Cochabamba (TI165); RS/Rio Grande do Sul/Brazil (TI24, TI27, TI28, TI131, TI156 residual foci).

Using data from Mahanalobis distances and Procrustes distances, we recovered two similarity dendrograms (Figure 8). The dendrograms retrieved with different data (Mahalanobis and Procrustes) show a similar topology. The dendrograms enable clear visualization of the distances between the study populations. Again, as in the previous results, the population of Argentina is relatively close to the populations of southern Brazil. In contrast, the populations of Bahia, Brazil, and Bolivia are also close.

### 4.2. Principal Component Analysis between Populations of Different Regions

**Figure 9:**
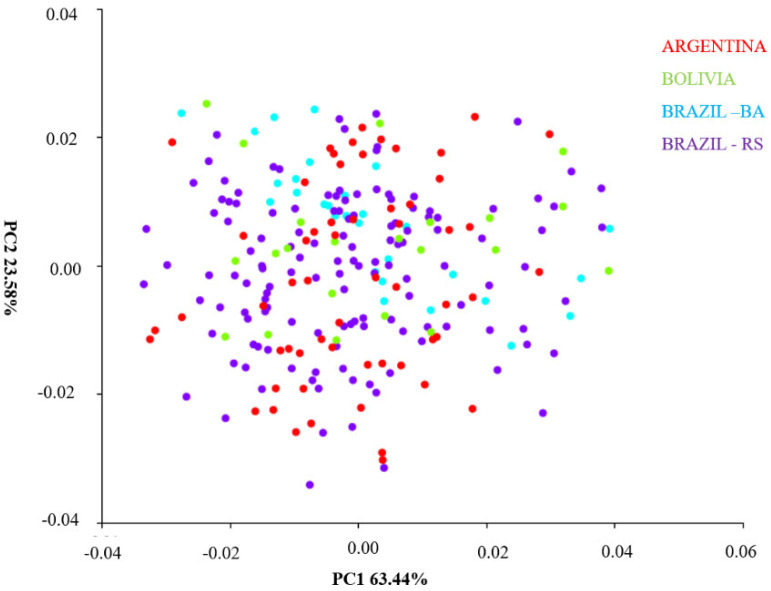
Principal component analysis (PCA) of *T. infestans* populations from different regions. TI24 (Guarani das Missões/RS/BR); TI25 (Argentina/Cordoba); TI26 (Argentina/Sta. Fe); TI27 (Entre Ijuís/RS/BR); TI28 (Sto. Cristo/RS/BR); TI131 (Sta. Rosa/RS/BR); TI156 (Sta. Rosa/residual foci/RS/BR); TI165 (Cochabamba/Bolívia); TI169 (Tremedal/residual foci/BA). BR-BRAZIL, RS-Rio Grande do Sul.

Principal component analysis (PCA) describes shape and size variables in a Cartesian plane (Figure 9). The first principal component (PC1) was responsible for 63.44% of the variations, and the second component explains 23.58%; together they accounted for 87.02% of shape variables in *T. infestans* populations from different regions (Figure 9). The disposition of the ellipses in the space of the Cartesian plane shows relative proximity of form in the heads of the populations of the studied regions; however, the population of Bolivia and Bahia presented greater capacity of differentiation in the x-axis. The populations of Argentina and Brazil (Rio Grande do Sul) maintained the approximate midpoint overlap (Figure 9).

### 4.3. Canonical Variance Analysis between Populations of Different Regions

**Figure 10:**
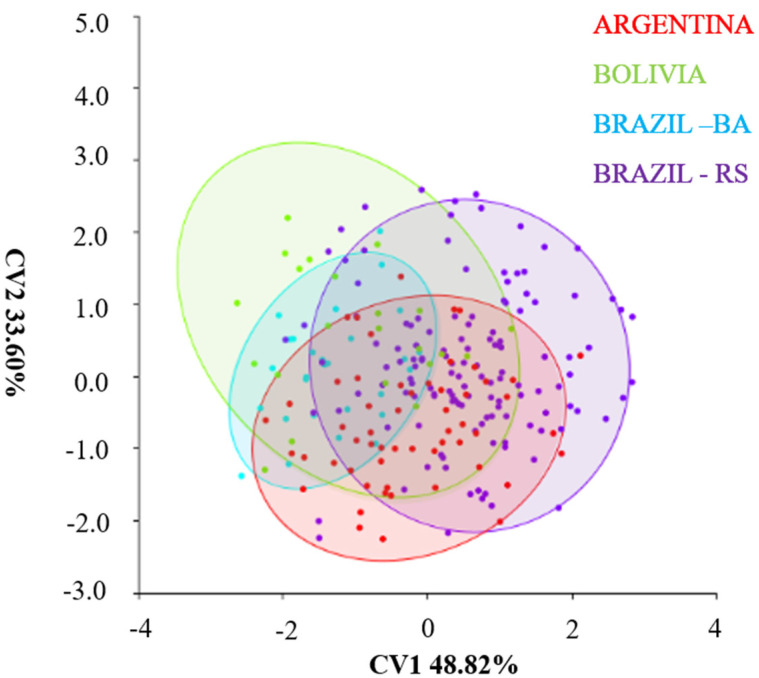
Exploratory analysis based on the shape variation of *T. infestans* heads from different regions after generalized analysis of Procrustes. Argentina (TI25, TI26) Bolivia (TI165), Bahia/ Brazil/ Residual foci 2010 (TI169); Rio Grande do Sul Brazil (TI24, TI 27, TI28, TI131, TI156).

The CVA was able to group the populations through the estimated multivariate correlation of the head of *T. infestans* populations. The analysis of canonical variables explained 100% of the shape variables. The first component (CV1) was responsible for 48.82% of the variations, and the second component (CV2) explained 33.6%; together they were responsible for 82.41% of the shape variations in *T. infestans* populations of different regions. The disposition of the ellipses in the space of the Cartesian plane shows a similarity of shape in the heads of the populations. The populations of Bolivia and Bahia (Brazil) maintain approximate midpoint overlap (Figure 10). In contrast, Argentina and Rio Grande do Sul also overlap in the space of the Cartesian axes (Figure 10).

## 5. Discussion

Despite the positive impact obtained from the reduction in *T. infestans* populations through vector control, this species still has epidemiological importance because of its ability to adapt to domestic and peridomiciliary environments [6,25]. However, for unknown reasons, *T. infestans* is still found in residual foci in the states of Bahia and Rio Grande do Sul, Brazil [26]. These episodic findings raise questions about the existence and persistence of residual foci in these regions, so our study sought to evaluate the morphometric relationship of *T. infestans* populations from residual foci and natural environments by geometric head morphometry.

Our results show that different ecotypic populations of *T. infestans,* and from different geographical sites, present significant morphometric differences, demonstrated through head geometric morphometry. We also show that macro-geographic patterns are better discriminated than micro-geographic patterns by geometric morphometry. The multivariate method has been shown to be useful in phylogenetic, systematic, or biogeographical studies with Triatominae [18,27,28,29,30,31].

Passive transport by humans is one of the main hypotheses to explain the distribution of *T. infestans* in South America [5]. In this way, it is possible that maintenance of residual foci of species such as *T. infestans* or *R. prolixus* in Brazil can be sustained by the passive transport of specimens from focal regions, where the wild environment or the lack of control keeps populations close to or in contact with human dwellings [5,6]. Our data demonstrate that Brazilian populations from Bahia and Cochabamba, Bolivia show few morphological differences. Similarly, populations from southern Brazil show greater morphometric proximity to populations from Argentina. The proximity of the border between the southern region of Brazil and Argentina is related to dispersion through the Argentinean Gran Chaco, as suggested by Piccinali et al. (2011) [10,11] and Waleckx et al. (2011) [9]. Even if our data do not support the hypothesis, the proximity between populations is demonstrated in this study.

In Brazil, so far, passive transport of *T. infestans* specimens has not been observed, as described in southern Chile [32], only the presence of residual foci in Bahia and Rio Grande do Sul. However, Brazil is Bolivia’s main economic market, as Bolivia exports 42% of its products to Brazil and, not unlike Argentina, has an active economic relationship with Brazil [33,34]. The results presented are not enough to trace the origins of the Brazilian residual populations, but all dynamics of the distribution of triatomines in the Americas support the need for investigations with other methods, such as phylogeographic studies, as in Campos-Soto [33], where the origin of insular species of *Mepraia* was described through phylogeographic studies that used molecular phylogenies, a molecular clock, and biogeography.

The Brazilian triatomine fauna is distributed by natural and artificial environments, the latter mainly associated with the transmission of Chagas to humans [35]. According to Forattini [5], maintenance of triatomines in artificial environments is mainly the result of anthropic action in natural environments. In addition to direct human actions on natural environments, secondary factors such as climate change can also significantly interfere with the radiation of insect vectors to new environments [36,37].

Triatomines are insects whose morphology is easily modeled by the environment. Rapid morphological changes in response to ecological factors can make specific identification difficult, as similar genetics but with marked morphological differences are common. [16] The *T. brasiliensis* complex is an interesting model to illustrate how morphology can obscure knowledge of the group’s species [16]. The *T. brasiliensis* species has a morphology shaped by ecotypic and environmental variations, as described in Kamimura [29]. Our studies support the phenotypic plasticity of *T. infestans* and show that geometric morphometry is useful to characterize morphological variation.

In this study, we used the PCA to characterize the variation in shape. The results show little variation in the shape of the heads, and length is the factor with the greatest potential for discrimination between populations of *T. infestans*. The CVA grouping method is consistent with the result of the PCA, showing little potential for discrimination between the studied populations. These methods are suitable for intra and interspecific studies [18,29]. However, the analyses of the CS and the Mahalanobis and Procrustes distances were useful to discriminate between the specimens, showing phenotypic variability of *T. infestans*.

Triatomines are excellent models to study phenotypic plasticity, as they are capable of rapidly modifying morphology in response to new habitats. Phenotypic plasticity is evident in triatomines. Our study shows, through head geometric morphometry, the morphometric variations in different populations of *T. infestans*. In addition to showing the phenotypic relationship between natural and residual populations of *T. infestans*, we point out the importance of new studies to confirm the relationships and origins between morphometrically close populations, as these data are important to elucidate the distribution dynamics of triatomines, as well as for collaborating in entomological control and surveillance.

## Figures and Tables

**Table 1:** Origin of *T. infestans* populations.

Species	ID	Locality	Initiate	Generation *
*T. infestans*	TI24	Guarani das Missões/RS */Brazil	2006	F22
*T. infestans*	TI25	Cordoba/Argentina	1996	F42
*T. infestans*	TI26	Santa Fé/Argentina	1994	F46
*T. infestans*	TI27	Entre Ijuís/RS/Brazil	1996	F42
*T. infestans*	TI28	Santo Cristo/RS/Brazil	1996	F42
*T. infestans*	TI131	Santa Rosa/RS/Brazil	2008	F18
*T. infestans*	TI156 ***	Santa Rosa/RS/Brazil	2014	F6
*T. infestans*	TI165	Cochabamba/Bolivia	2015	F4
*T. infestans*	TI169 ***	Tremedal/BA **/Brazil	2010	F14

* RS—Rio Grande do Sul; ** BA—Bahia; *** residual foci.

**Table 2:** Mahalanobis distances between *T. infestans* populations from different localities.

Samples	TI25	TI26	TI169	TI165	TI131	TI156	TI24	TI27	TI28
TI25	0.00								
TI26	1.3641	0.00							
TI169	3.7685	1.9871	0.00						
TI165	3.6017	2.2425	1.735	0.00					
TI131	3.2075	3.7394	3.885	3.3427	0.00				
TI156	3.4669	2.1847	2.3269	2.0245	2.6227	0.00			
TI24	1.6277	1.6703	4.132	4.219	3.840	3.896	0.00		
TI27	3.5218	2.4463	2.472	2.2997	3.4285	1.2399	3.736	0.00	
TI28	3.7424	1.9028	2.1189	1.6677	2.3642	1.3464	4.2309	2.0298	0.00

TI24 (Guarani das Missões/RS/BR); TI25 (Argentina/Cordoba); TI26 (Argentina/Sta. Fe); TI27 (Entre Ijuís/RS/BR); TI28 (Sto. Cristo/RS/BR); TI131 (Sta. Rosa/RS/BR); TI156 (Sta. Rosa/residual foci/RS/BR); TI165 (Cochabamba/Bolívia); TI169 (Tremedal/Residual foci/BA).

**Table 3:** Mahalanobis distances between populations of *T. infestans* from different regions.

Regions	1	2	3	4
1. ARG	0.0000			
2. BOL	1.7701	0.0000		
3. BR_BA	1.5817	1.5082	0.0000	
4. BR_RS	1.2438	1.9315	1.9828	0.0000

ARG (Argentina); BOL (Bolivia); BR_BA (Brazil/Bahia/residual foci); BR_RS (Brazil/Rio Grande do Sul).

## Data Availability

The database used and/or analyzed during the current study is available from the corresponding author on reasonable request.
